# Multilevel analysis of facial expressions of emotion and script: self-report (arousal and valence) and psychophysiological correlates

**DOI:** 10.1186/1744-9081-10-32

**Published:** 2014-09-26

**Authors:** Michela Balconi, Maria Elide Vanutelli, Roberta Finocchiaro

**Affiliations:** 1 Research Unit in Affective and Social Neuroscience, Department of Psychology, Catholic University of the Sacred Heart, Milan Largo Gemelli, 1, 20123 Milan, Italy

**Keywords:** Facial expression of emotion, EMG, Valence, Psychophysiology

## Abstract

**Background:**

The paper explored emotion comprehension in children with regard to facial expression of emotion. The effect of valence and arousal evaluation, of context and of psychophysiological measures was monitored. Indeed subjective evaluation of valence (positive vs. negative) and arousal (high vs. low), and contextual (facial expression vs. facial expression and script) variables were supposed to modulate the psychophysiological responses.

**Methods:**

Self-report measures (in terms of correct recognition, arousal and valence attribution) and psychophysiological correlates (facial electromyography, EMG, skin conductance response, SCR, and heart rate, HR) were observed when children (N = 26; mean age = 8.75 y; range 6-11 y) looked at six facial expressions of emotions (happiness, anger, fear, sadness, surprise, and disgust) and six emotional scripts (contextualized facial expressions). The competencies about the recognition, the evaluation on valence and arousal was tested in concomitance with psychophysiological variations. Specifically, we tested for the congruence of these multiple measures.

**Results:**

Log-linear analysis and repeated measure ANOVAs showed different representations across the subjects, as a function of emotion. Specifically, children’ recognition and attribution were well developed for some emotions (such as anger, fear, surprise and happiness), whereas some other emotions (mainly disgust and sadness) were less clearly represented. SCR, HR and EMG measures were modulated by the evaluation based on valence and arousal, with increased psychophysiological values mainly in response to anger, fear and happiness.

**Conclusions:**

As shown by multiple regression analysis, a significant consonance was found between self-report measures and psychophysiological behavior, mainly for emotions rated as more arousing and negative in valence. The multilevel measures were discussed at light of dimensional attribution model.

## Introduction

In the last two decades, developmental psychology has seen an increasing interest in the study of emotion comprehension. Specifically, emotional face recognition and understanding represent a primary social competence, because they contribute to social interactions and social management [1]. These competencies are related to general cognitive functions and it was shown that language was the most important predictor of nonverbal emotion recognition ability [2]. Indeed both gender and verbal skills are important predictors of children’s emotional awareness [3].

More specifically, *Bullock and Russell*[4] proposed a model in which children acquire a system to represent and classify emotions which is based on a limited number of wide categories. The most important of them are the two dimensional axes of the hedonic value and the arousal level. This model was tested by some empirical studies which found that firstly children interpret facial expressions in terms of pleasure-displeasure (bipolar hedonic value) and intensity (arousal level). Only successively they use more articulated and wider conceptual categories [5,6]. To verify the type of categorization applied to the emotional domain, affective responses organized around the arousal and valence dimension include subjective experience, often measured using self-report responses to affective stimuli. At this regard, Self-Assessment Manikin (SAM) was used to test this subjective emotional correlates [7]. It was also demonstrated that age, facial expression intensity and emotion category are important for predicting accuracy on emotion-processing tasks [8].

Previous results demonstrate how task type and children’s mood influence children’s emotion processing [9]. Indeed, in order to explain this developmental process, the type of emotions children have to recognize is a first main factor related to decoding competencies [10]. More generally, in line with Russell’s model of emotional experience, emotion fundamentally varies activation in centrally organized appetitive and aversive motivational systems that have evolved to mediate the wide range of adaptive behaviors necessary for an organism struggling to survive in the physical world [11-13]. Most pleasant affects are held to be associated with the appetitive motivation system; unpleasant affects with defensive motivation [14]. Thus, a primary distinction among emotional events is whether they are appetitive or aversive, positive or negative, pleasant or unpleasant, which clearly relates to the motivational parameter of direction. Secondly, all agree that hedonically valenced events differ in the degree of to which they arouse or engage action, which is related to intensity parameter. Emotional intensity probably reflects the strength of activation in motivational systems subserving appetitive and defensive behaviors and, as such, has impact on the type of physiological response. Intensity was conceptualized as “predator imminence”, or the distance of the threatening pattern from the subject [15] or in terms of distance from an aversive or appetitive goal [16]. More generally, arousal in humans appears to reflect the degree to which a stimulus elicits appropriate appetitive or defensive behaviors. Thus, the two dimensions of pleasure and arousal explain the majority of the emotional experience and subjective judgment, and the increased perception of emotional significance of a stimulus in term of valence may increase the perception of its arousing power [17].

Secondly, it should be noted that young subjects showed to be competent in the decoding of the primary or simple emotions (e.g. happiness and anger), but they have more difficulties in processing secondary or complex emotions, such as pride and embarrassment [18-20]. To identify these emotions more time and more informational cues must be analyzed. Moreover, as regard to the secondary emotions or emotions developed only later during the developmental phases, a more accentuated difficulty in understanding causal antecedents (the events that caused the emotion expressed by face) and contextual relations (the social situations in which the emotion is produced) emerges [21]. *Bormann-Kischkel et al.*[10] observed a specific difficulty in understanding the emotions that present a lack of correspondence between people expectations and environment events. These emotions have an external and social origin, such as surprise, dismay, and astonishment. In parallel, *Capps, Yirmiya, and Sigman*[20] observed a greater impairment in recognizing and labelling the expression of those emotions that have an external locus of control and, simultaneously, that require a wide knowledge of the social scripts and of their social consequences. In line with this hypothesis, *Baron-Cohen, Spitz, and Cross*[22] suggested that the comprehension is more difficult for emotions that imply the activation of some cognitive functions, such as mentalization and metarepresentation. In general, previous results provide evidence for late developmental changes in emotional expression recognition with some specificity in the time course for distinct emotions [23]. Indeed it was found that some emotions, like disgust and sadness, are more often confused with other primary and early developed emotions, such as anger or fear and they are not spontaneously labelled in comparison with other emotions such as anger, fear or happiness.

Thus, it was also supposed that, through a progressive process of script generalization, a “situated” comprehension of emotions arises. The process of emotion categorization is well illustrated by the use of adequate attribution. Indeed, emotional labels constitute the final step of a developmental process that goes through a “dimensional attribution” (characterized by the presence of pleasure-displeasure correlate) to a “situational attribution” (the script representation). This should be taken into consideration in studying the development of emotional decoding, because these competencies seem to be bound not only to cognitive but, above all, to social and communicative competencies, which have an influence on emotion conceptualization. Thus, another main concern is represented by contextual and situational elements that cause emotion and that might facilitate or not the emotion processing and comprehension [24]. As *Russell and Widen*[25] underlined, in everyday experience children use facial expressions in order to infer emotions. On the other hand, the facial cues are always located in an interactive context. In other words, it is necessary to take into account the role of a wider socializing context. Therefore, the concept of emotional context, considered as a complex and multidimensional representation of situational events, is relevant in facial expression processing.

Thirdly, it was observed that behavioral and physiological responses to emotional pictures co-vary with system evaluation (pleasure/displeasure) and motive intensity (arousal) [26,27]. Psychophysiological responses are not directly observable, and they include cardiovascular, electrodermal, respiratory measures, etc. It was underlined that emotion comprehension plays a critical role in adaptive behavior, since it promotes survival and guides human behavior by exerting a direct influence on brain responsiveness and psychophysiological activities. Between the others, facial action revealed by EMG measure (electromyogram), heart rate, and skin conductance were observed to variate in concomitance of pleasure and displeasure reports while viewing of emotional patterns.

More specifically, about the psychophysiological measures, facial behavior using electromyography (EMG) suggested they were sensitive to the valence dimensions, with increased corrugator activity in response to unpleasant patterns and zygomatic activity in response to pleasant patterns. Facial EMG (electromyographic) activity accompanies changes in appetitive (positive) and defensive (negative) activation [28]. Specifically, the corrugator muscle appears to be responsive of to judgment of unpleasant event compared to neutral pictures [27]. Many studies found a consistent and significant relationship between corrugator and hedonic valence, with greater corrugator activity elicited when viewing the most unpleasant stimuli [29]. Moreover, *Bradley, Codisposti, Sabatinelli, and Lang*[30] showed that pictures that produce disgust (for example mutilation), that were higher in arousal, prompt larger changes than other unpleasant pictures.

Other physiological measures of emotional behavior include heart rate (HR), with observed increased HR acceleration to pleasant patterns and increased HR deceleration to unpleasant patterns [30]. Moreover, investigations exploring cardiovascular activity in emotion perception assessed variations as a function of differences in stimulus intensity, as this variable was revealed critical in eliciting orienting or defense response [27,30-32]. Low-intensity stimuli were found to relate with heart rate deceleration, whereas intense stimuli were observed to activate defense responses associated with heart rate acceleration [33-36]. Nevertheless, also contrasting results were collected, since heart initially decelerated, rather than accelerated, when people viewed pictures of unpleasant emotional events, contrary to the notions that these aversive stimuli might prompt defensive heart rate acceleration [27,30,37]. However, different experimental paradigms were adopted in previous research and, in some cases, no direct comparison can be conducted between them.

Moreover, it was found electrodermal activity (Skin Conductance Response, SCR) consistently varies with emotional intensity, with larger responses elicited in either unpleasant and pleasant context and that are more pronounced in those that are rated as highly arousing [27,38,39]. Thus, also electrodermal reactions increase with increases in defensive or appetitive activation [30,37]. In general, it was found increased skin conductance when people view pictures rated as emotional, compared to neutral, regardless they are rated pleasant or unpleasant in hedonic valence [27]. However, when hedonic valence and emotional arousal were co-varied, skin conductance responses were largest for highly arousing stimuli, irrespective of hedonic valence [40], consistent with the notion that these reactions primarily reflect differences in emotional arousal, rather than hedonic valence per se.

About these psychophysiological variations in response to emotions and facial stimuli, an important debate regards the presence of a coherent response by psychophysiological measures in childhood, as it was observed in adult behavior. Previous research found consistent patterns of psychophysiological activation also by children in response to emotional stimuli [41,42]. Nevertheless, to verify the coherence of these physiological measures in young people in response to facial emotional patterns, specific analysis should be conducted which included both arousal and valence parameters.

Therefore, emotional behavior manifests within multiple domains, comprehending conceptual and self-report attribution, autonomic responses (physiological systems), and the comprehension of contextual components, which all may have a significant role in this process. However, no previous study has directly analyzed the relationship between these multilevel measures, that is self-report evaluation based on valence and arousal parameters, psychophysiological behavior and contextual cue variability. The present study was finalized to explore the convergence of these different measures.

In the present research the effect of some main factors, valence modulation (emotional type) from one hand, and contextual effect (face alone vs. facial display within a script), from the other, was considered. Specifically, we explored their influence on physiological reactivity (autonomic activity) and emotional attribution (self-report attributional process), which are all relevant to the description of the emotional responses [26,43]. Thus, the purpose of this study is to verify the attended psychophysiological and attributional responses to emotion variation, and, secondly, to show that the attributional process was related to valence and to context modulation.

Previous assumptions should be strengthened by the following hypotheses:

1) Faces evaluated as more negative or positive in term of valence and arousing power should elicit more intense responses, being the subjects more engaged with the stimulus, whereas neutral stimuli should be less involving and intense, and, consequently, differ in affective rating from emotional stimuli. The interaction effect of these two parameters (i.e. valence and arousal) is also expected. This would suggest that effects due to emotional arousal should be greater for highly unpleasant and pleasant stimuli, which were rated as slightly more arousing than stimuli evaluated as less positive/negative [44].

2) Secondly, HR, EMG, and SCR should show a modulation in correlation with emotionally relevant, arousing and pleasant or unpleasant stimuli. We expected that subjects might be more emotionally involved by a highly negative or positive and more arousing stimulus than neutral or low-arousing pictures, and that they might have a more intense psychophysiological activation while viewing a negative or positive than a neutral pattern when they are also perceived as more arousing [13]. This should produce an increased SCR and HR response, and the modulation of facial EMG. Specifically, we expected an increased SCR for more positive and negative emotions, an increased corrugator activity in response to negative emotions and an increased zygomatic activity in response to positive emotions. Finally a general higher HR should be expected mainly for more arousing emotions.

3) Furthermore, we expect that children may have more difficulties to decode and understand emotions generally considered as more complex and learned only successively (such as disgust) rather than primary basic emotions (such as happiness, anger, and fear). In particular, we focus our attention on the representation of the dimensional axes of hedonic/arousal value, that engenders the acquisition of a more complex conceptual representation [5,45]. Thanks to this acquisition it can be produced the developmental process, that includes an initial competence in the discrimination of basic emotional categories and a successive comprehension of more complex emotional categories (as disgust or sadness). Thus we supposed that about these emotions children could have more difficulty to give a spontaneous attributional correct attribution (in term of valence and arousal) to the facial patterns. Secondly they should be less physiologically responsive to these emotional cues, based on the intrinsic relationship that we expected to exist between attributional and psychophysiological processes.

4) Fourthly, based on the “situational” perspective to explain facial emotion comprehension, we may suppose that emotion decoding is the result of the elaboration of multiple emotional cues, among which facial patterns (facial expressions), behavioral correlates (the causal bonds between events), as well as specific contextual factors (eliciting emotional context). The comparison between two different types of condition (only a facial expression of an emotion; a facial expression within an emotional script) allows us to explore in detail the role of the eliciting context in the emotion. We suppose that script facilitates subjects’ recognition. According to our hypothesis, a script will function as a facilitation cue to correctly interpret the whole emotional event. This facilitation should be mainly more evident for the secondary emotions, as disgust, because in order to comprehend this emotion, subjects have to understand some contextual or “external” elements. Finally, this facilitation effect should be supported by psychophysiological measures, and in parallel situational cues should support the SCR increasing (more positive and negative emotions); the increased corrugator activity in response to negative emotions, and the increased zygomatic activity in response to positive emotions.

## Method

### Participants

The sample includes 26 normal children. Ages varied from 6 to 11 (*M* = 8.75; S.D. = 0.78; range = 6-11.5; 15 females and 11 males). None of them presented cognitive or linguistic deficits. With regard to cognitive competencies, children presented a middle-high or high functioning cognitive profile (WAIS-IV FSIQ: *M* = 87; range: 70-120). No history of psychiatric or neurological impairments was observed for the participants. Indeed two neuropsychologists applied a specific semi-structured interview before the experimental session to test no clinical impairments. The presence of other deficits on the perceptive or cognitive levels was excluded. Child’ parents gave informed written consent to participate in the study by their sons, and the research was approved by the local ethics committee (Catholic University Ethic Committee, Department of Psychology).

## Materials

### Facial stimuli

**Figure 1:**
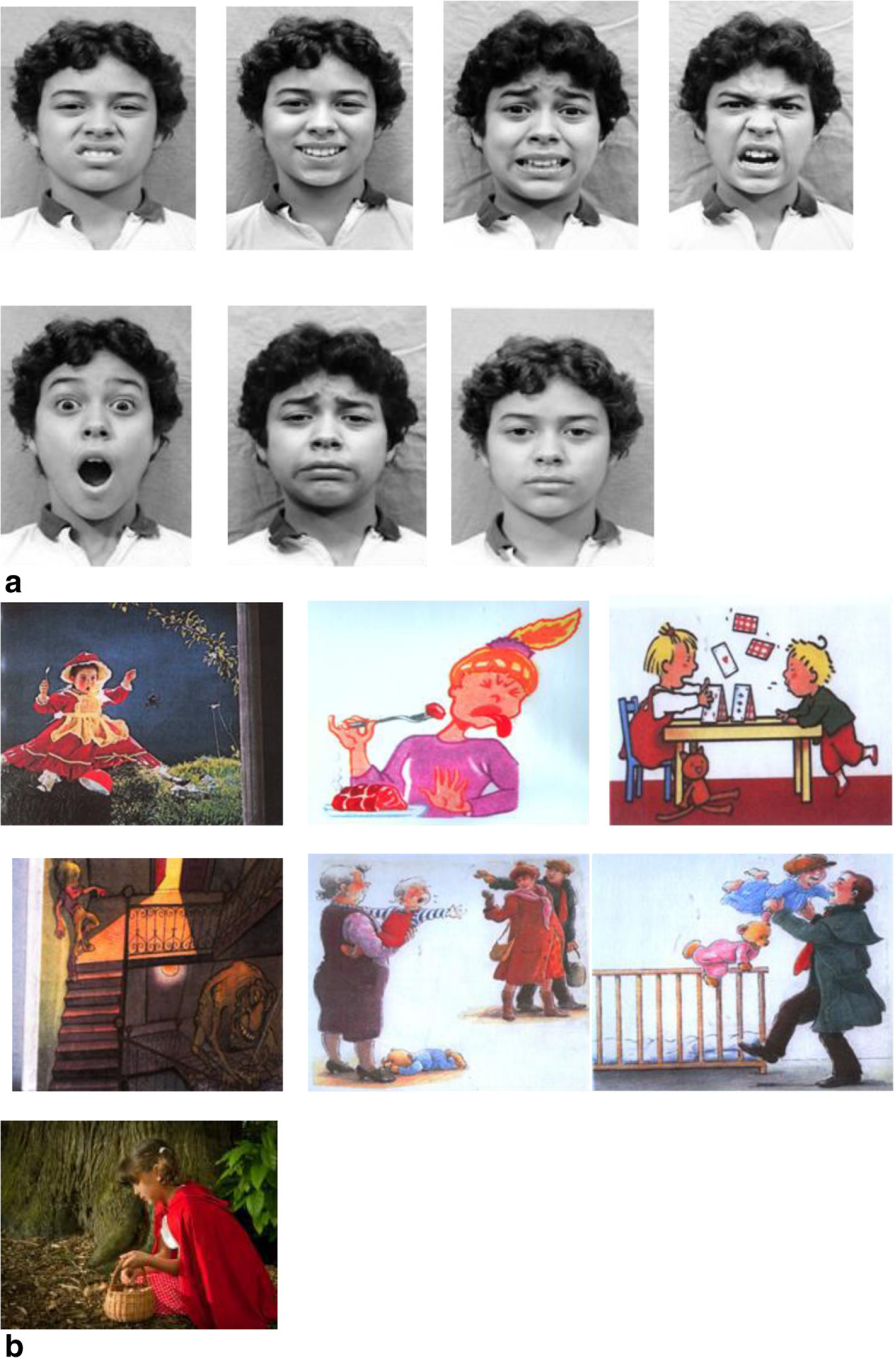
Examples of (a) facial stimuli and (b) emotional scripts.

The facial stimuli (cardboards black and white 10 cm × 10 cm), which consist of an emotional face of a young boy showing six emotions (happiness, sadness, anger, fear, disgust and surprise) and one neutral face. The stimulus material was selected by *Ekman and Friesen* database [46]. We have opted for a young actor aged similarly to the experimental subjects, in order to facilitate the identification process, which would make easier the recognition task (Figure 1a).

### Emotional scripts

The material consists of 6 pictures (coloured cardboards 10 cm × 15 cm) with an emotional content and one neutral picture (see Figure 1b). Pictures illustrate contextualized situations eliciting the emotional correlates of happiness, sadness, anger, fear, surprise and disgust [5,45]. In particular each picture presents a character (a girl or a boy) in an interactive context (with peers or adults). In addition, the presence of a clear emotional facial expression was considered a discriminant stimulus for the selection of the pictures. The pertinence of the emotional content for each emotional script, the homogeneity of the stimuli in terms of cognitive complexity and familiarity were tested in a pre-experimental phase (12 males and females; 6-11 years). Stimulus homogeneity, intended as the degree of difficulty in comprehending the situation represented in the script (clarity of the context and the represented subjects), and the complexity (number of details represented) were tested with a 5-points Likert scale. No significant differences were found between emotions for homogeneity *F* (6,11) = 1.18, *p* = .40; and complexity: *F* (6,11) = 1.64, *p* = .31).

In each phase, first time stimuli were presented simultaneously, in order to allow familiarization with the material. In a second assessment, they were presented one at time, in a random sequence, varying the order of the stimulus across the participants. Furthermore, to avoid a possible order effect between the experimental conditions, some subjects were submitted to face decoding condition and successively to emotional script condition, whereas other subjects decoded the stimulus materials in an opposite sequence (firstly the emotional script and then the facial expression).

### Procedure

Subjects were told that they had to evaluate some pictures (faces or scenes) based on some rating scales. Self-Assessment Manikin was used to test the self-report measures on a nine-point scale hedonic value (positive/negative) and arousal value of the emotional content (more/less arousing) [7]. After each presentation of the stimulus (stimulus presentation duration = 15 sec.) subjects were invited to evaluate it, no longer viewing the image. During stimulus presentation subjects’ psychophysiological responses were acquired. Furthermore, through a half-structured interview grid [47], the experimenter invited the child to observe the stimulus set and to describe the emotional feelings represented (“What is that facial expression?”). It was made another focal question about the situation illustrated by the pictures (“What happened?”). Interviews were audio- and video-taped and scored verbatim. Three judges examined the verbal material encoded, in order to analyze specific conceptual categories relatively to the correctness of the verbal labels (correct recognition). For the first level of analysis, a correct answer included an explicit emotional label (such as “happiness”) or synonymous terms (“joy”) [47].

### Psychophysiological recording procedure

#### SCR, HR and EMG data reduction

Skin conductance response was measured continuously with a constant voltage by Biofeedback (Biofeedback 2000, version 7.01). Before the attaching electrodes, the skin was cleaned with alcohol and slightly abraded. SCR was recorded from two electrodes placed on the medial phalanges of the second and third finger of the non-dominant hand. The sample rate was of 400 Hz. SCRs elicited by each stimulus were scored manually and defined as the largest increase in conductance in a time window from 1,500 to 4,000 ms after stimulus presentation (for the procedure see *Amrhein, Muhlberger, Pauli, & Wiedermann*) [48]. Trials with artifacts were excluded from analysis, whereas trials with no detectable response were scored as zero. The electrocardiogram was recorded using electrodes on the left and right forearms. Inter-beat intervals of the HR were converted to heart rate in beats per minute, to detect HR modulation during viewing stimuli. Trials with artifacts were excluded from analysis, whereas trials with no detectable response were scored as zero. Facial electromyographic (EMG) activity in the zygomaticus major and corrugator supercilii muscle regions were considered. The electrodes (4 mm diameter Ag/AgCl electrodes), filled with Surgicon electrolyte paste, were positioned over the corrugator and zygomatic muscles in accordance with guidelines for psychophysiological recording [49,50]. Frequencies of interest generally ranged from 20 to 400 Hz. Corrugator and zygomatic EMG responses were successively scored as the difference between the mean rectified corrugator/zygomatic signals present during the presentation of the stimuli and the mean rectified signals in the 1 s prior to stimulus presentation (baseline measure). A positive value indicates that the corrugator/zygomatic measures were greater during the experimental phase than during the baseline phase. All the data were acquired for the time interval of stimulus presentation (15 sec.) and successively normalized.

The exact synchrony between the stimulus presentation and the psychophysiological data acquisition was guaranteed by the introduction of specific marker by a second experimenter, simultaneously to the onset of the stimulus presentation. A successive analysis of the video-taped registration of the entire experimental session furnished other checking of this synchrony.

## Analysis and results

### Self-report measures

The statistical analysis applied to self-report measures included two steps: a first step, where log-linear analysis was applied to correctness of emotional evaluation; a second step, where repeated measure ANOVAs was applied. Type I errors associated with inhomogeneity of variance were controlled by decreasing the degrees of freedom using the Greenhouse-Geiser epsilon.

A log-linear hierarchical analysis (saturated model) was applied to subject labeling (correct labelling of emotion) with factors correctness (correct/incorrect, 2) × condition (face/script, 2) × emotion (type, 7) variables (see *Areni, Ercolani, Scalisi*) [51] (Table 1). In both conditions (emotional face and script), the emotions were largely recognized by the subjects. In fact, they correctly labeled each emotion (with increased correct recognition more than incorrect, *χ*^2^(1, N = 26, 11.38, p ≤ .01) independently from the type of task *χ*^2^(1, N = 26, 1.21, p = .30). However, emotional type showed significant effect *χ*^2^(1, N = 26, 8.03, p ≤ .01). Post-hoc comparisons (standardized residuals) revealed that anger, fear, surprise and happiness were better recognized than disgust, sadness and neutral faces (all comparisons p ≤ .01).

**Table 1:** Self-report measure of correctness (percentage), arousal and valence for each emotion and condition (face and script)

**Self-report rating**	**Anger**		**Fear**		**Suprise**		**Happiness**		**Disgust**		**Sadness**		**Neutral**	
*Face*	M	(sd)	M	(sd)	M	(sd)	M	(sd)	M	(sd)	M	(sd)	M	(sd)
Correctness	89	1.34	91	2.87	84	1.89	80	2.09	69	1.98	65	1.56	64	1.22
Arousal	8.46	0.56	8.52	1.09	8.11	0.77	7.09	0.65	6.32	0.65	4.55	0.54	3.91	0.65
Valence	2.33	0.98	2.11	0.78	2.87	0.22	8.04	3.50	3.50	0.76	2.33	0.64	4.98	0.68
*Script*	M	(sd)	M	(sd)	M	(sd)	M	(sd)	M	(sd)	M	(sd)	M	(sd)
Correctness	86	2.33	88	3.98	85	2.87	77	2.09	74	1.98	67	2.09	60	2.09
Arousal	8.40	0.78	8.16	0.86	8.09	0.49	7.32	0.36	6.98	0.65	4.13	0.53	3.08	0.54
Valence	2.39	0.65	2.66	0.71	2.43	0.76	8.76	0.65	2.98	0.84	2.38	0.67	4.55	0.39

SAM rating nine-points (valence: 1 = high negative, 9 = high positive; arousal: 1 = low, 9 = high).

About the valence attribution, ANOVA showed a significant emotion (F(6, 25) = 10.30, p ≤ .01, ɳ^2^ = .38) and emotion × condition effect (F(6, 25) = 9.14, p ≤ .01, ɳ^2^ = .38) (Table 1). Post-hoc comparisons (contrast analysis, with Bonferroni corrections for multiple comparisons) showed increased negative valence attribution for anger, fear, surprise and sadness in comparison with happiness and neutral face, as well as happiness was considered as more positive than the other faces (all comparisons p ≤ .01). Moreover, it was found a more negative attribution for disgust in the case of script more than face condition F(1, 25) = 10.79, p ≤ .01, ɳ^2^ = .40). No other comparison was statistically significant (all comparisons p ≥ .01).

About the arousal attribution it was found a significant emotion (F(6, 25) = 10.15, p ≤ .01, ɳ^2^ = .39) and emotion × condition effect (F(6, 25) = 9.56, p ≤ .01, ɳ^2^ = .37). Post-hoc comparisons showed increased arousal attribution for anger, fear, and surprise in comparison with happiness and sadness (all paired comparisons p ≤ .01). Moreover all the emotional faces were considered more arousing than neutral faces (all paired comparisons p ≥ .01). In addition, about the interaction effect, disgust was found as more arousing in the case of script than face condition F(1, 25) = 8.09, p ≤ .01, ɳ^2^ = .33). No other comparison was statistically significant (all paired comparisons p ≥ .01).

### Psychophysiological measures

Successively, repeated measure ANOVAs, with two independent repeated (within-subjects) factors (condition × emotion), were applied to each dependent measure (SCR; HR; EMG).

#### SCR

**Figure 2:**
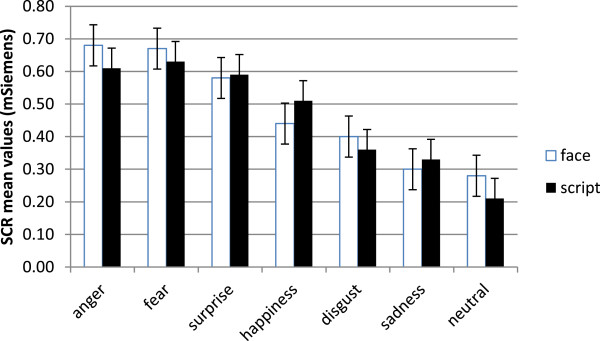
Mean (and SE) SCR modulations in response to different emotions.

ANOVA showed significant main effect of emotion (F(6, 25) = 9.56, p ≤ .01, ɳ^2^ = .37). As shown by contrast effects, anger, fear and surprise revealed increased SCR in comparison with happiness, sadness, disgust and neutral stimuli. Moreover, disgust and happiness showed higher SCR than neutral faces (all comparisons p ≤ .01) (Figure 2).

#### HR

**Figure 3:**
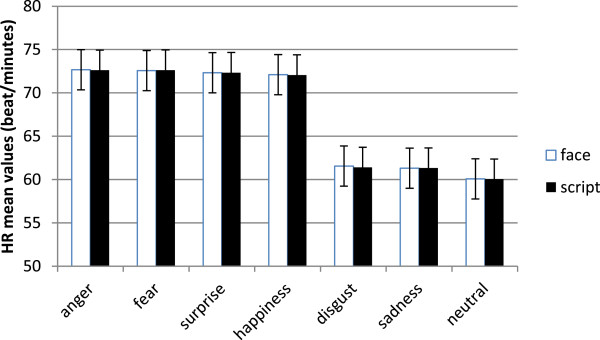
Mean (and SE) HR modulations in response to different emotions.

ANOVA showed significant main effect of emotion (F(6, 25) = 10.98, p ≤ .01, ɳ^2^ = .40). As shown by contrast analyses, anger, fear surprise and happiness revealed increased HR in comparison with sadness, disgust and neutral stimuli. Moreover, disgust and sadness showed increased HR than neutral faces (all comparisons p ≤ .01) (Figure 3).

#### EMG

**Figure 4:**
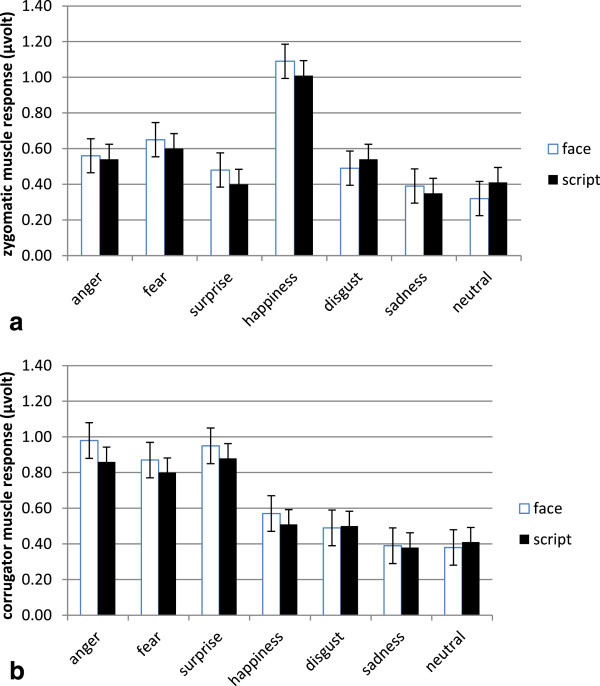
Mean (and SE) (a) zygomatic and (b) corrugator modulations in response to different emotions.

Zygomatic EMG activity revealed significant differences as a function of emotion (F(6, 25) = 10.76, p ≤ .01, ɳ^2^ = .41). As shown by contrast effects, EMG activity was enhanced in response to positive stimuli in comparison with negative and neutral faces (all comparisons p ≤ .01). Contrarily, corrugator EMG activity was increased for negative emotions, respectively anger, fear, and surprise in comparison with happiness, disgust, sadness and neutral stimuli (all comparisons p ≤ .01) (Figure 4 and 4b).

### Regression analysis between valence and arousal ratings and psychophysiological measures

Distinct stepwise multiple regression analyses were performed for each psychophysiological measure and emotion, considering the mean values for face and script condition. Predictor variables were arousal and valence ratings, and predicted variables were EMG, SCR, and HR amplitude for each emotion. We report in Table 2 the cumulative multiple correlations between predictor and predicted variables (R), cumulative proportion of explained variance (R^2^), and the regression weights (β) for the regression equation at each step of the multivariate analysis.

**Table 2:** Stepwise multiple regressions

	**Anger**		**Fear**		**Surprise**		**Happiness**		**Sadness**		**Disgust**		**Neutral**	
**Predictor**	**Arousal**	**Valence**	**Arousal**	**Valence**	**Arousal**	**Valence**	**Arousal**	**Valence**	**Arousal**	**Valence**	**Arousal**	**Valence**	**Arousal**	**Valence**
**Model**	**1**	**2**	**1**	**2**	**1**	**2**	**1**	**2**	**1**	**2**	**1**	**2**	**1**	**2**
*Zygomatic*														
R	0.13	0.28	0.20	0.34	0.22	0.33	0.44	0.76	0.18	0.30	0.26	0.37	0.14	0.26
R2	0.01	0.07	0.04	0.09	0.04	0.11	0.19	0.57	0.03	0.09	0.06	0.03	0.01	0.06
β	0.20	0.21	0.24	0.23	0.15	0.11	0.34	0.28	0.15	0.18	0.23	0.26	0.23	0.20
std error	0.21	0.22	0.15	0.17	0.21	0.28	0.18	0.27	0.23	0.20	0.17	0.19	0.18	0.26
t	1.02	0.87	0.95	0.88	0.78	0.70	1.76^*^	1.54^*^	0.77	0.84	0.96	0.87	0.67	0.59
*Corrugator*														
R	0.49	0.64	0.1 = 51	0.69	0.35	0.52	0.24	0.41	0.18	0.29	0.22	0.40	0.18	0.29
R2	0.24	0.40	0.26	0.47	0.12	0.27	0.05	0.18	0.03	0.07	0.04	0.18	0.03	0.07
β	0.31	0.32	0.23	0.21	0.27	0.20	0.23	0.28	0.32	0.38	0.36	0.29	0.20	0.23
std error	0.25	0.20	0.20	0.18	0.18	0.19	0.33	0.34	0.30	0.28	0.22	0.27	0.17	0.20
t	1.88^*^	1.03	1.93^*^	1.02	1.43^*^	0.99	1.02	0.96	0.65	0.49	0.90	0.78	0.56	0.43
*SCR*														
R	0.43	0.69	0.54	0.72	0.38	0.62	0.32	0.59	0.21	0.38	0.29	0.51	0.17	0.29
R2	0.18	0.47	0.27	0.51	0.13	0.41	0.38	0.34	0.04	0.14	0.07	0.25	0.03	0.08
β	0.18	0.28	0.22	0.20	0.18	0.23	0.29	0.20	0.34	0.35	0.35	0.57	0.27	0.33
std error	0.11	0.17	0.28	0.26	0.20	0.21	0.21	0.30	0.30	0.30	0.26	0.23	0.22	0.29
t	1.80^*^	1.19^*^	1.97^*^	1.07	1.65^*^	1.17^*^	1.12^*^	1.15^*^	0.88	0.63	1.10^*^	1.08^*^	0.54	0.45
*HR*														
R	0.42	0.70	0.50	0.80	0.44	0.71	0.36	0.65	0.22	0.38	0.35	0.67	0.18	0.29
R2	0.17	0.49	0.25	0.64	0.19	0.50	0.14	0.42	0.04	0.14	0.12	0.36	0.03	0.08
β	0.17	0.20	0.20	0.22	0.28	0.27	0.29	0.27	0.19	0.34	0.25	0.22	0.39	0.28
std error	0.22	0.26	0.18	0.15	0.20	0.21	0.32	0.30	0.15	0.18	0.18	0.29	0.27	0.22
t	1.84^*^	1.14^*^	1.98^*^	1.18^*^	1.90^*^	1.09^*^	1.12^*^	1.10^*^	0.67	0.78	1.55^*^	1.12^*^	0.77	0.60

Arousal and valence as predictor variables, pshychophysiological measures as predicted variables.

*= P≤.05.

As shown in Table 2, arousal and valence accounted for the amplitudes of zygomatic muscle for happiness, whereas mainly arousal rating accounted for corrugator muscle for anger, fear and surprise. In addition, valence and arousal explained the HR (increasing) more for anger, fear, surprise, and happiness. Finally, SCR increased response was mainly explained by the two predictors for anger, fear, and surprise, and secondly for disgust and happiness.

## Discussion

The present study produced three major results, that we summarize in the following points. First, there was a clear differentiation in children’ conceptualization (in terms of arousal and valence) as a function of different emotions; besides, the psychophysiological measures were highly modulated by emotional types, and arousal and valence parameters accounted for the psychophysiological variations in relationship with different emotional patterns; finally the presence of two different types of task – a facial expression decoding and a script comprehension –induced significant differences in the subjective representations only for a limited number (mainly disgust) of emotions.

For the first time we used multimodal measures to explore the evaluation effect (based on valence and arousal) on psychophysiological behavior taking into account an ample range of emotions. Secondly we applied this multimodal approach to study the specific domain of facial expression of emotions whereas other previous research did not specifically consider this emotional domain. Thirdly we considered the facial expression of emotion with and without an emotional script context to study the contextual impact on face decoding. Therefore the situated perspective was adopted in the present research.

As hypothesized by the dimensional approach to emotion [52,53], the representation of the emotional domain was based on a conceptual space defined by two exes, arousal and hedonic value. In particular, the emotions with a high arousal level and a negative value were better understood, if compared with other emotions. Specifically, the emotions of fear, anger and surprise were well recognized and well labeled. A significant higher arousing power was attributed to them, and these emotions were also considered as more negative. Moreover, they were better recognized than the other emotions, specifically in comparison with sadness and disgust. The positive emotion of happiness was considered as less arousing and more positively valenced and it was well represented and recognized. On the contrary, disgust appears to be more difficult to be identified, as well as sadness, and they both were considered as less arousing and less negative. It should be considered that in present research we opted to evaluate the ability of subjects in spontaneously labelling the face/script they saw. As revealed, disgust and sadness were not immediately labelled, but in many cases they were correctly described (using a semi-structured interview) only after a successive enquire. Therefore, the subjects showed a general ability in recognizing the two emotions, although this recognition was less immediate. It should be based on the increased complexity to decode these emotions, because they are learned only successively in comparison with other primary emotions (such as anger and fear).

Therefore a first main result of the present study was that the dichotomy pleasure/displeasure and high/low arousal was considered relevant by the subjects, confirming a significant role in emotion representation, as indicated by previous researches [19,53,54]. In fact, not only the hedonic category was systematically well represented, but it was correctly identified in terms of negativity or positivity. Moreover, arousal rating can be considered a predictive cue of the ability to classify and differentiate emotional correlates. Indeed, it was correctly used when the child was able to attribute an adequate label to the emotion, while when the child cannot conceptualize the emotion, the arousal value seems to be more ambiguous (for example for disgust) or less relevant (sadness).

As regard to more negative and arousing emotions (fear, anger and surprise) some recent study [55,56] revealed high rates of recognition, that the researcher attributes to the central adaptive function of these negative high arousing emotions. Indeed, they has a main role for the individual safeguard, both on an ontogenetic and a phylogenetic level. They may be represented as a cue in order to detect unfavorable environmental conditions [19,54]. Accordingly to the functional model [57,58], the emotional expressions represent a response to a particular event, significant in terms of costs and benefits for people. Specifically, the expression of anger and fear represents the perception of a threat for the personal safeguard and, therefore, it requires a greater investment of attentional resources. The prominence of specific category of emotion (more negative and arousing) may suggest their central role in emotion acquisition in comparison with other less relevant (and less arousing) emotions in childhood.

The script condition introduces another main explicative factor, regarding the emotional representation. Indeed, the presence of a specific context generally does not affect the correctness of the emotional label attribution, but it produces a discriminant effect exclusively for one emotion, that is disgust. Indeed in presence of a specific situational context disgust was better characterized in terms of arousal (more clearly arousing) and valence (more negatively valenced). The presence of the interactional features that characterize the emotional experience seems to introduce a facilitation element for emotion comprehension, also producing a better description in the emotion labeling (more correct recognition). It was possible to state that the situational component constitutes a facilitation cue, because it allowed the subjects to activate a more complex conceptual representation, which takes into account the context in which the emotional event happens, the emotional causes, the logical order of actions and their consequences [4].

It was noticeable, however, that the script enables a wider and a more complete representation only in case of a this “secondary” emotion, which maximally has a benefit from the situated condition. It was observed that emotion recognition was allowed by the development and the generalization of an emotional script, that is, a child can recognize a specific emotion by verifying the presence of several prototypical elements that are arranged in precise causal and temporal sequences. These scripts include not only facial expressions, but also the representation of causal factors, physical and social context, several actions and their consequences, as well as the cognitive appraisal of the situation and the subjective experience [4]. Among these cues, the representation of the causal bonds, that is a set of causal events and of their behavioral consequences, has a remarkable significance, because they constitute the more explicative elements of the emotional experience [5,45,59].

To conclude, even if our study does not allow us to state which of the two representational modalities (facial pattern comprehension or script decoding) precedes the other, it was possible to observe that the situational correlates provide a facilitation cue for the representation of emotional correlate when a secondary emotion is represented. However, no specific facilitation effect was observable in case of “primary” emotions, which were well recognized and described also in absence of contextual cues.

A relevant main result of the present research was related to the psychophysiological measures which were shown to vary in concomitance to the type of stimuli (different emotions) and to the categorization process (the subjective ratings). In fact, subject revealed a coherent psychophysiological behavior in response to the emotions, independently from the condition (script or face). Moreover, it was shown that SCR, HR and EMG were modulated as a function of the two main axes of valence and arousal, as they were rated by the subjects.

Firstly, SCR was shown to be increased when children processed emotional faces and scripts rated as high arousing and negative (anger, fear and surprise), whereas it decreased in concomitance with stimuli rated as low arousing (mainly sadness, disgust, and neutral patterns). A similar profile was observed for HR, which showed higher values in case of more positive, more negative and arousing stimuli. These results were in line with many other studies on adults, which postulated a significant HR effect for more arousing and relevant stimuli [33-36]. Moreover, the variation in term of arousing power (high vs. low) may determine the different impact of the emotional cues, since perception of a high arousal generally induces a consistent HR increasing independently from the stimulus valence. These multiple parameters and their combination were relevant to comprehend the effect of emotions on psychophysiological data.

An important result was also observed for the facial EMG values. Indeed we found that children were highly responsive to facial stimuli and scripts, by adopting a sort of “facial feedback” modality, since they used similar facial configurations displayed by the pictures (consonant behavior) [60]. It was observed an increasing of mimic activity in case of some conditions: the different emotions evoked distinct facial EMG response patterns, with increased zygomatic muscle activity to positive patterns and increased corrugator muscle activity to negative patterns, whereas both the corrugator and the zygomatic muscle response patterns were less pronounced in sadness, disgust and neutral condition. More generally, corrugator muscle activity was increased in response to more negative and arousing stimuli, mainly for fear, anger, and surprise. In addition, as revealed by regression analysis, arousal parameter showed to explain in greater measure the corrugator modulation, whereas valence was less relevant to describe the psychophysiological activity in response to negative, highly arousing patterns. Contrarily, zygomatic muscle was modulated by both arousal and valence, with significant increasing responsiveness related to happiness.

These variations may mark a psychophysiological response in case of a high arousing situations, since relevant (with arousing power) stimuli seem to produce and reinforce a coherent psychophysiological behavior. Contrarily, subject reported a reduced arousing power for sadness and partially for disgust, fact that may explain the concomitant reduced EMG, SCR and HR values. Thus, more arousing conditions showed a perfect consonance between subjective evaluation and psychophysiological (both facial and autonomic) measures. Specifically, anger, fear, surprise and happiness were rated as more emotionally involving. In parallel, the psychophysiological behavior was responsive of this subjective self-evaluation, with an increased “positive” (zygomatic) facial expression and a higher autonomic activity (increased HR) for happiness, from one hand; an increased “negative” (corrugator) facial expression and higher arousal response (more SCR and HR) for anger, fear and surprise, from the other.

However, more generally the modulation of psychophysiological measures was mainly related to arousing power more than to valence, since independently from the valence, the stimuli rated as high arousing (anger, fear, surprise and happiness) were able to induce a more significant and coherent emotional response. Regression analysis confirmed these results: mainly arousal attribution was significant to determine the psychophysiological variations, able to explain SCR, HR and facial response modulation, since subjects “shared” the facial behavior and autonomic activity observed in both positive vs. negative conditions.

Thus, in general psychophysiological measures may be interpreted as functional mechanism of “mirroring” the emotional condition displayed by the facial stimuli, where “sharing” similar emotional responses allows a direct form of understanding and recognize emotion by a sort of simulation process. More specifically, contexts evaluated as emotionally involving and significant may ingenerate a consonant shared response by the observer, who firstly recognizes and secondly “mimic” (by face and autonomic behavior) the somatic markers related to the experienced emotions [61]. Moreover, based on these results we may suggest that the gradual development of emotional competencies proceeds from more basic and simple emotions, which are primarily acquired by children, to more complex and less prominent emotions, which might be less relevant in terms of salience. Brain correlates may support this different learning process, related to a “maturation effect” which might explain more deeply the early acquisition of the recognition abilities in response to more salient and relevant emotions in term of human safeguard and the successive acquisition for the less relevant (less threatening and primary for the safeguard) emotions.

To summarize, self-report measures were replicated by psychophysiological behavior, that showed to vary coherently in relationship with different emotions. Children revealed a consonant and adequate behavior in terms of labeling (correct recognition), evaluation (valence and arousal attribution) and psychophysiological responsiveness. However, a clear advantage was observed for some specific emotions, those rated as more arousing and negative (fear, anger and surprise). It was suggested these emotions may be central to people safeguard and they may be priority developed by children. Arousal attribution was considered as the most critical parameter to explain the emotion recognition process and the psychophysiological behavior. Contrarily, sadness and disgust were less prominent in terms of both arousal and valence, and in some cases they were also less correctly recognized. The contextual cues (script condition) may allow to perform a better attribution, mainly for the emotion of disgust. In case of more complex emotional cue, the context (script) contribution was relevant to complete the correct recognition.

However, about the main limitations of the present study, future research may explore more directly the intrinsic effect induced by facial expression of emotion taking into account also gender effect. Indeed previous research found significant differences between male/female children in response to the emotional type. Secondly, the arousal effect we found in the present study should be better considered in relationship with different emotional valence taking into account a wider range of facial expressions which may cover the ample orthogonal axes low/high arousal positive/negative valence. Thirdly, due to the limited sample we used for the present research, it is crucial to integrate the present data with an ampler sample size, in order to extend the present results to a general population.

## Competing interests

The authors declare that they have no competing interests.

## Authors’ contributions

MB planned the experiment, supervised the experiment; designed the statistical analysis, wrote the paper. RF realized the experiment; applied the analysis; provided the editorial and reference assistance. MEV realized the experiment; applied the analysis; provided the editorial and reference assistance. All authors read and approved the final manuscript.
